# Factors influencing computed tomography determined stone-free rates after ureteroscopy in real-world and its impact on retreatment rates at medium-term follow-up

**DOI:** 10.1007/s00345-025-05721-2

**Published:** 2025-06-03

**Authors:** Diana M. Lopategui, Ansh Bhatia, Joao G. Porto, Aravindh Rathinam, Jean C. Daher, Ryan R. Chen, Haikel Haile, Mariam Ahumada, Maggie Meyreles, Jonathan Katz, Robert Marcovich, Hemendra N. Shah

**Affiliations:** 1 Desai Sethi Urology Institute, University of Miami, Miller School of Medicine, Miami, USA; 2 Department of Interventional Radiology, University of Miami, Miller School of Medicine, Miami, USA; 3 Desai Sethi Urology Institute, 1120 NW 14th St #2107, 15th Floor, Miami, FL 33136 USA

**Keywords:** Ureteroscopy, Stone free rate, Non-contrast computed tomography scan, Residual stone fragments, Retreatment. Nephrolithiasis, Holmium laser, Laser lithotripsy

## Abstract

**Background and objectives:**

Residual stone fragments (RFs) following ureteroscopy increase the risk of reintervention. This study assesses stone-free rates (SFR) using non-contrast computed tomography (NCCT), identifies factors influencing SFR, and investigates the relationship between RFs and retreatment rates (RTR).

**Methods:**

Patients who underwent ureteroscopy for urolithiasis between September 2017 and March 2024 were included if they had postoperative NCCT. Exclusion criteria include nephrocalcinosis and combined intrarenal surgery. Clinical data, procedural details, and postoperative outcomes were analyzed. Univariate and multivariate Cox regression models assessed factors affecting SFR and RTR. A Kaplan Meier curve analyzed time to retreatment after surgery.

**Key findings and limitation:**

Among 457 patients (519 renal units) the true SFR was 42.8%, increasing to 58.4% and 78.6% when RFs < 3 mm and < 4 mm were included. Factors negatively associated with SFR included prior urolithiasis treatment, percutaneous nephrolithotomy, positive urine culture, prior stenting, larger stone size, and mid- or lower-pole stone location. Conversely, exclusively ureteric stones and single stones were associated with higher SFR. On multivariate analysis, positive urine culture, prior urolithiasis treatment, increasing stone size, and mid-pole stone location remained significant predictors of reduced SFR. Logistic regression revealed the odds ratio for RTR with RF > 3 mm versus RF < 3 mm was 7.14 (95% CI: 1.96–24.39). Limitations included the risk of missing some stone-related outcomes during follow-up.

**Conclusions and clinical implications:**

The NCCT determined true SFR was 42.8%, with the size of RFs strongly correlating with the RTR. Patients with residual calculi should be counselled about increased risk of retreatment rate.

## Introduction

Kidney stones are a common and debilitating condition, posing an increasing burden on the U.S. healthcare system. From 1988 to 1994 to 2007–2010, self-reported prevalence rose significantly, reaching 10.6% in men and 7.1% in women, a 70% increase across all demographics [1]. The economic impact has also escalated, with direct treatment costs nearly doubling from $4.5 billion in 2000 to $9 billion in 2021, alongside $775 million in annual indirect costs [2]. Residual stone fragments (RFs) are a critical concern due to their strong association with regrowth, symptomatic passage, and reintervention. A commercial insurance database analysis revealed that 23% of patients undergoing ureteroscopy required retreatment within one year, incurring an additional $16,369 per patient. Similarly, unplanned follow-ups after ureteroscopy added $23,436 in healthcare costs [3–5]. RFs are the primary cause of these complications, contributing significantly to patient morbidity and healthcare burden [6]. Thus, achieving a true stone-free status is essential for optimizing outcomes post-ureteroscopy. Unfortunately, there is no clear definition of stone-free rate (SFRs). Reported stone-free rates after ureteroscopy often exceed 90% but are predominantly based on radiography or ultrasound, which have lower sensitivity than postoperative non-contrast computerized tomography (NCCT), leading to potentially overestimating the SFRs [7–10].

This study primarily aims to determine SFR after ureteroscopy using NCCT imaging and to evaluate various factors influencing these SFR. Our secondary aim is to assess the relationship between post-ureteroscopy RFs and unplanned reinterventions. Additionally, we reviewed the literature on SFR after ureteroscopy assessed with an NCCT scan. By addressing these issues, we aim to provide data that can help in appropriate patient counseling and lay a foundation for developing strategies to improve patient outcomes.

## Materials and methods

### Patients

Following institutional review board approval (20180511), we retrospectively reviewed patients > 18 years who underwent ureteroscopy for urolithiasis at a tertiary academic center between September 2017 and March 2024. The study included all ureteroscopy cases, regardless of stone burden. Patients with stones > 2 cm unsuitable for percutaneous nephrolithotomy (PCNL) were informed about the potential need for two-stage ureteroscopy, with SFR assessed after the planned second procedure. Only patients who underwent post-operative NCCT were included. Patients with chronic nephrocalcinosis or combined endoscopic intrarenal surgery were excluded, but those undergoing ureteroscopy for RF removal after PCNL were included. Patients with congenital renal anomalies were not excluded from this study.

### Procedure

Urine cultures were obtained three weeks before surgery, and patients with positive results were treated with culture-specific antibiotics. Surgeries were performed under general anesthesia in the lithotomy position. An 11/13 ureteral access sheath (UAS) was routinely used, while a 12/14 UAS was preferred for pre-stented patients. If the UAS could not be advanced, the ureteroscope was maneuvered over a sensor wire. Gravity irrigation with warm saline supplemented by a Pathfinder^®^ (Utah Medical Products Inc, Utah, USA) or Single-Action Pump (Boston Scientific, Massachusetts, USA) was used. Stone fragmentation was performed using a 200-micron fiber with a holmium laser (60 W, 100 W, or Moses™ 120 W). Dusting settings were predominantly attempted at setting of 0.3 J and 30–40 Hz, while subsequent fragments were further fragmented at 0.5–0.8 J and 5–10 Hz. Attempts were made to retrieve all stone fragments using a zero-tip basket (Boston Scientific, Massachusetts, USA) or an N-gage basket (Cook Medical, Indiana, USA). These were then sent for chemical analysis. Postoperative ureteral stents were placed unless the ureter was pre-stented. All procedures were conducted outpatient, with same-day discharge following a brief period of observation.

### Postoperative management

Patients were advised to undergo a low dose NCCT scan with slice thickness of 3 mm at 3–4 weeks post-ureteroscopy, with residual stone burden defined by the largest fragment’s maximum dimension, measured by a single urologist (HNS). Stent removal was performed at home by pulling the string (5–7 days post-placement) or in the clinic within 3–4 weeks via cystoscopy after reviewing SFR on NCCT. For RF > 4 mm, a second ureteroscopy was offered. Patients opting for observation underwent renal ultrasound at 6-month intervals post-ureteroscopy. All patients were encouraged to complete a stone risk profile two months after stent removal and received dietary and medical management guidance to prevent recurrence. Regardless of RF presence, all patients were advised to have an annual renal ultrasound to monitor stone recurrence or growth.

### Data collection

Preoperative demographic variables included age, sex, comorbidities, and stone-specific factors such as location, size, and multiplicity were assessed. Preoperative NCCT findings were reviewed for stone characteristics and anatomic abnormalities. Intraoperative data included surgery duration, defined as the total time spent by the patient in the operation room and laser energy use. Each side kidney and/or ureter was considered a single renal unit. Postoperative NCCT scans were analyzed to assess SFRs and RF size. Postoperative parameters included stone composition and retreatment during the available follow-up period.

### Data analysis

Univariate and multivariate Cox proportional hazards regression models assessed the association between predictor variables and the SFR. In the univariate analysis, each predictor variable was individually tested for its association with SFR. Variables with a *p*-value < 0.05 in the univariate analysis were considered statistically significant. The initial multivariate model included variables that were significant in the univariate analysis. Cox regression was used to assess factors that contribute to retreatment. Model Assessment: the assumptions of the Cox proportional hazards regression model were assessed using graphical methods and diagnostic tests. Proportional hazard assumptions were evaluated using Schoenfeld residuals. The incidence rate ratio (IRR) for a specific independent variable quantifies the change in the incidence rate (count) associated with a one-unit increase in the predictor variable while keeping other variables constant. An IRR > 1 signifies an increase in the rate, whereas an IRR < 1 reflects a decrease. Notably, an IRR = 1 indicates no change in the incidence rate. Factors affecting retreatment were calculated using logistic regression. A Kaplan Meier curve was created using the time to retreatment from index surgery. Statistical analyses were performed using R version 4.2.3 (Boston, MA, USA).

## Results

During the study period, 457 patients with a mean age of 57.1 years underwent ureteroscopy for urolithiasis and met the inclusion criteria. Since 62 patients underwent bilateral same session ureteroscopy, 519 renal units were included in the study (Table 1). True SFR (defined by no RF on postoperative NCCT scan) was achieved in 42.8% of renal units. When the definition of overall SFR was expanded to include fragments < 2 mm, < 3 mm, < 4 mm, and *≤* 5 mm, the SFR increased progressively to 44.9%, 58.4%, 69.4%, and 80%, respectively. Analyzing SFR by stone location revealed an SFR of 64.1% for renal stones, 82% for ureteric stones, and 53.9% for stone burdens spanning both the kidney and ureter when SFR was defined as fragments < 3 mm.

**Table 1:** Baseline demographics, intraoperative and post-operative outcome

Variable	Value
**Baseline demographic**	
**Mean age** (years)	57.1 ± 14.1
**Sex**	
Male	285 (62.4%)
Female	172 (37.6%)
**Mean Body Mass Index** (kg/m²)	28.95 ± 6.03
**Comorbidities**	
Diabetes Mellitus	105 (23.0%)
Antiplatelets/Anticoagulants use	100 (21.8%)
**Prior Treatment for Stones**	197 (43.1%)
ESWL	78 (17.1%)
Ureteroscopy	100 (21.9%)
PCNL	46 (10.0%)
**Anomalous Kidney**	34 (6.55%)
**Prior ureteral stent in-situ (renal units)**	148 (28.5%)
**Stone details (per renal unit)**	
**Mean preoperative Stone Size** (mm)	13.53 ± 8.10
**Stone location**	
Only kidney	304 (58.6%)
Only ureter	139 (26.8%)
Kidney and ureter	76 (14.7%)
**Stone location in kidney #**	
Lower pole	247
Mid- pole	128
Upper pole	116
Renal pelvis	82
**Number of stones**	
Single	216 (41.6%)
Multiple	303 (58.4%)
**Intraoperative details**	
**Operative time (min)**	98.1 ± 58.8
**Mean Laser energy used (KJ)**	44 ± 22
**Predominant stone composition per patient**	
Calcium oxalate	362 (79.2%)
Calcium phosphate	17 (3.3%)
Uric acid	62 (13.6%)
Struvite	16 (3.5%)
**Stone free rate on NCCT scan by residual fragment size per renal units**	
0 mm	222 (42.8%)
≤ 2 mm	233 (44.9%)
≤ 3 mm	303 (58.4%)
< 4 mm	360 (69.4%)
< 5 mm	415 (80.0%)

**Fig. 1:**
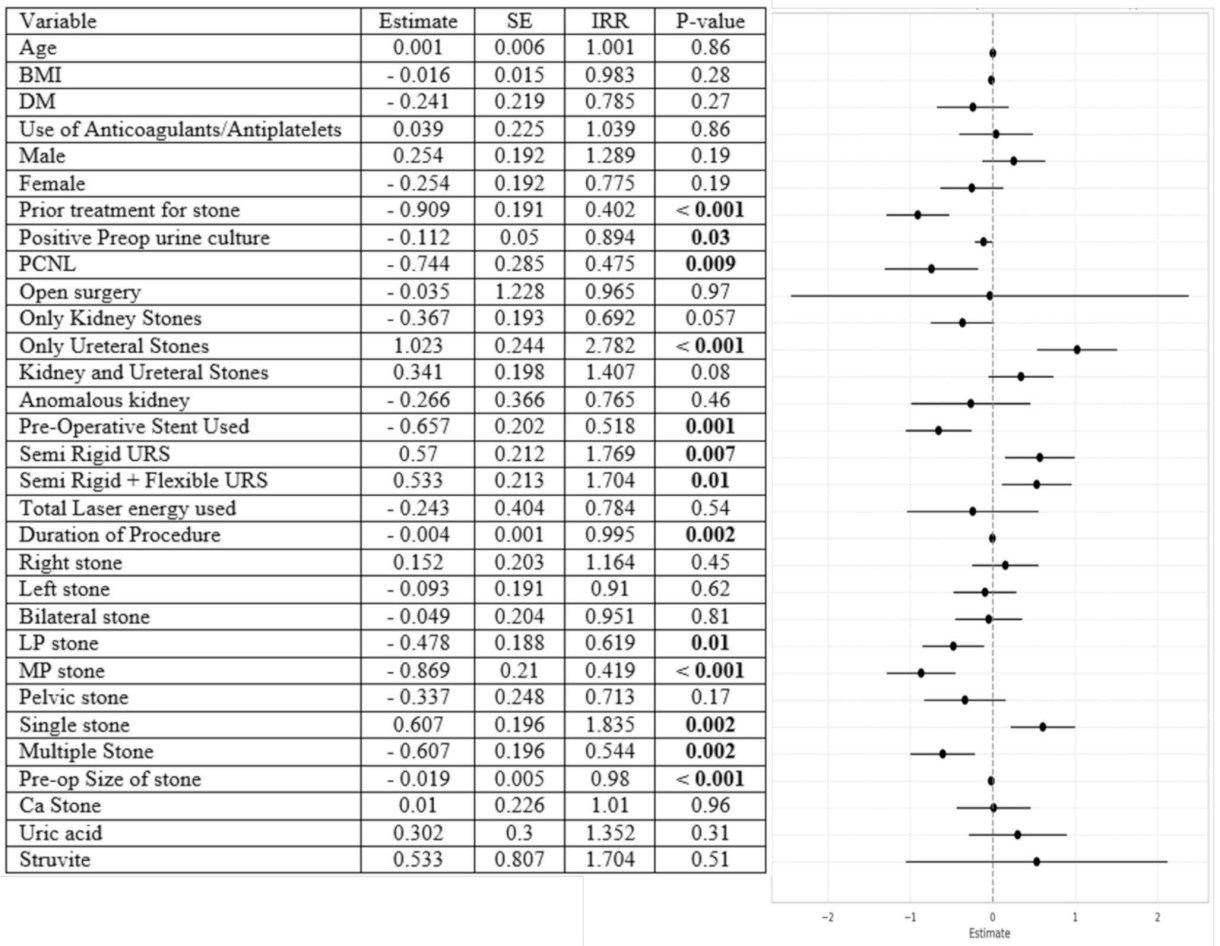
Univariate regression analysis of variables affecting stone free rate (SFR) defined as < 3 mm on postoperative NCCT) (BMI - body mass index; DM - diabetes mellitus; PCNL - percutaneous nephrolithotomy; LP - lower pole; MP - mid-pole; preop - preoperative; URS - ureteroscopy; ca - calcium oxalate stone)

**Fig. 2:**
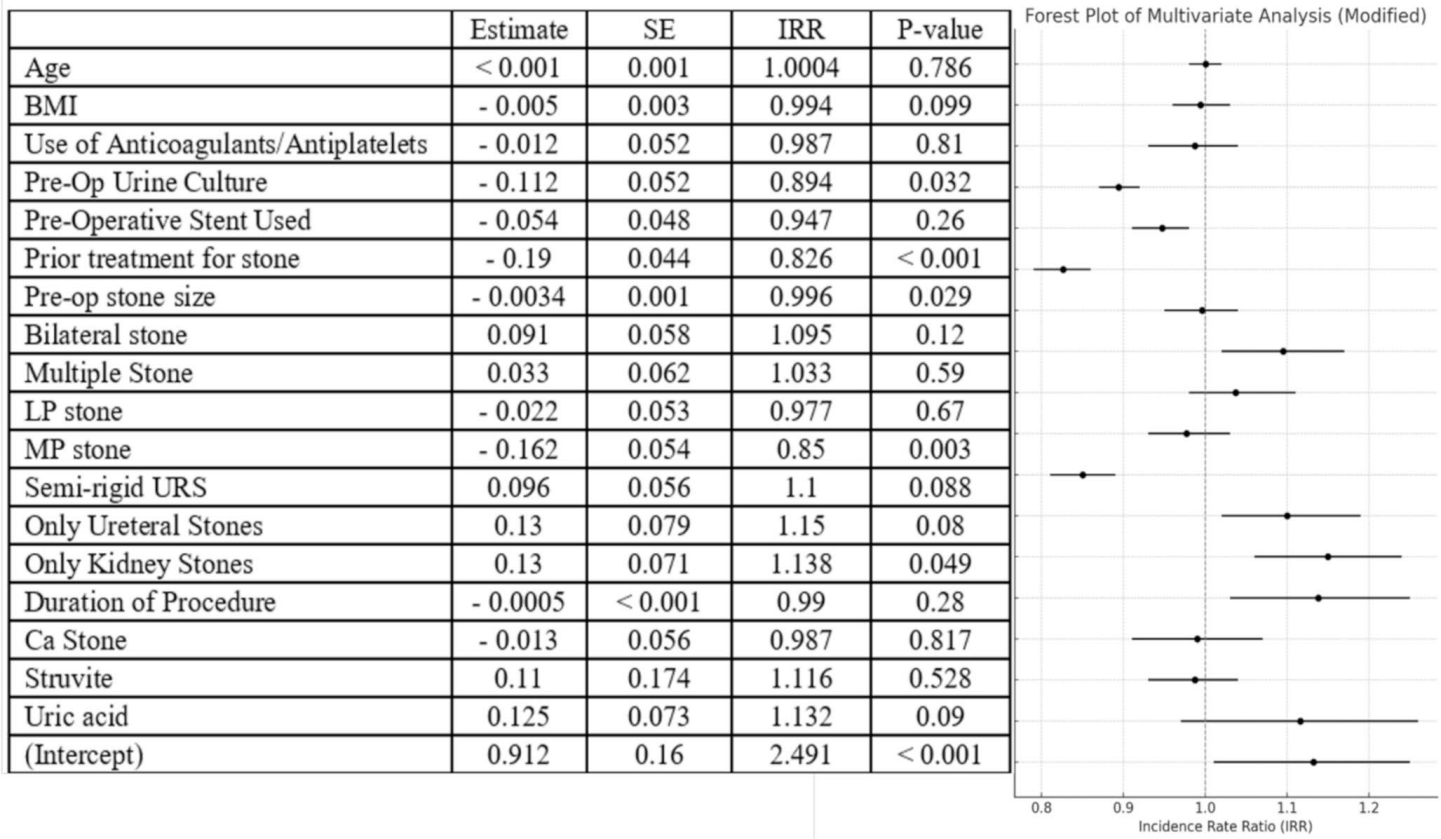
Multivariate regression analysis of variables affecting stone free rate (SFR) defined as < 3 mm on postoperative NCCT) (BMI - body mass index; LP - lower pole; MP - mid-pole; preop - preoperative; URS - ureteroscopy; ca - calcium oxalate stone)

We conducted a univariate regression analysis after defining SFR as cases with RF < 3 mm maximum dimension. This revealed a significant reduction in SFR among patients with a prior history of urolithiasis treatment (*p* < 0.001), previous PCNL (*p* < 0.001), positive preoperative urine culture (*p* = 0.03), or pre-stenting before ureteroscopy (*p* < 0.001). Additionally, larger stone sizes (*p* < 0.001) and stones located in the lower (*p* = 0.01) or mid-pole (*p* < 0.001) were associated with reduced SFR (Fig. 1). Conversely, factors associated with significantly higher SFR included the presence of stones only in the ureter (*p* < 0.001) and the presence of a single stone (*p* = 0.002) (Fig. 1). On multivariate analysis, decreased SFR was independently associated with positive preoperative urine culture (*p* = 0.03), a prior history of urolithiasis treatment (*p* < 0.0001), increasing stone size (*p* = 0.02), and mid-pole stone location (*p* = 0.003) (Fig. 2).

**Fig. 3:**
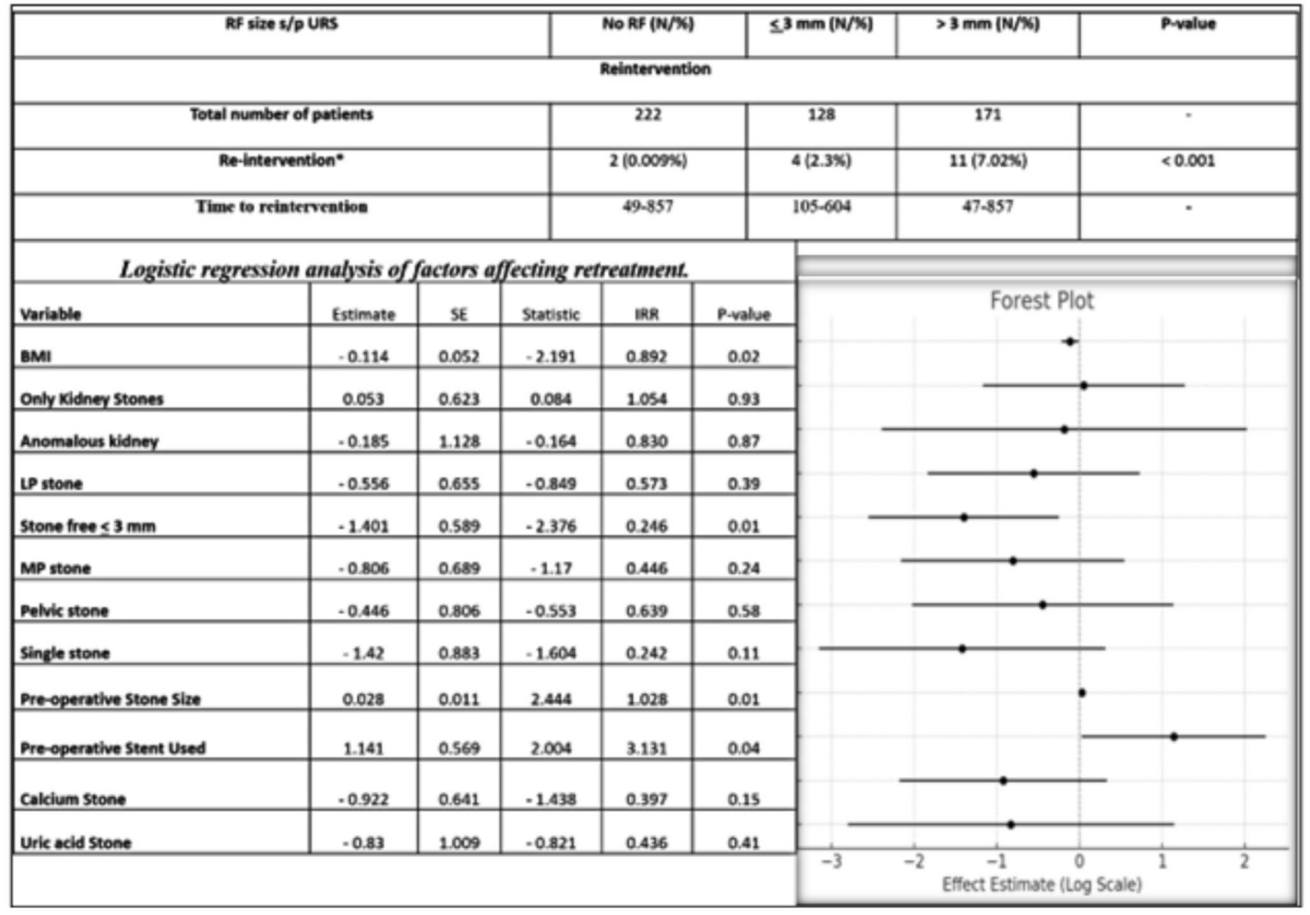
Retreatment rate based on size of residual fragments and logistic regression analysis of variables affecting retreatment rate for RF as defined as > 3 mm on postoperative NCCT (mean follow-up of 26 months (IQR: 12.6–36 months; range: 1–70 months); BMI - body mass index; LP - lower pole; MP - mid-pole)

**Fig. 4:**
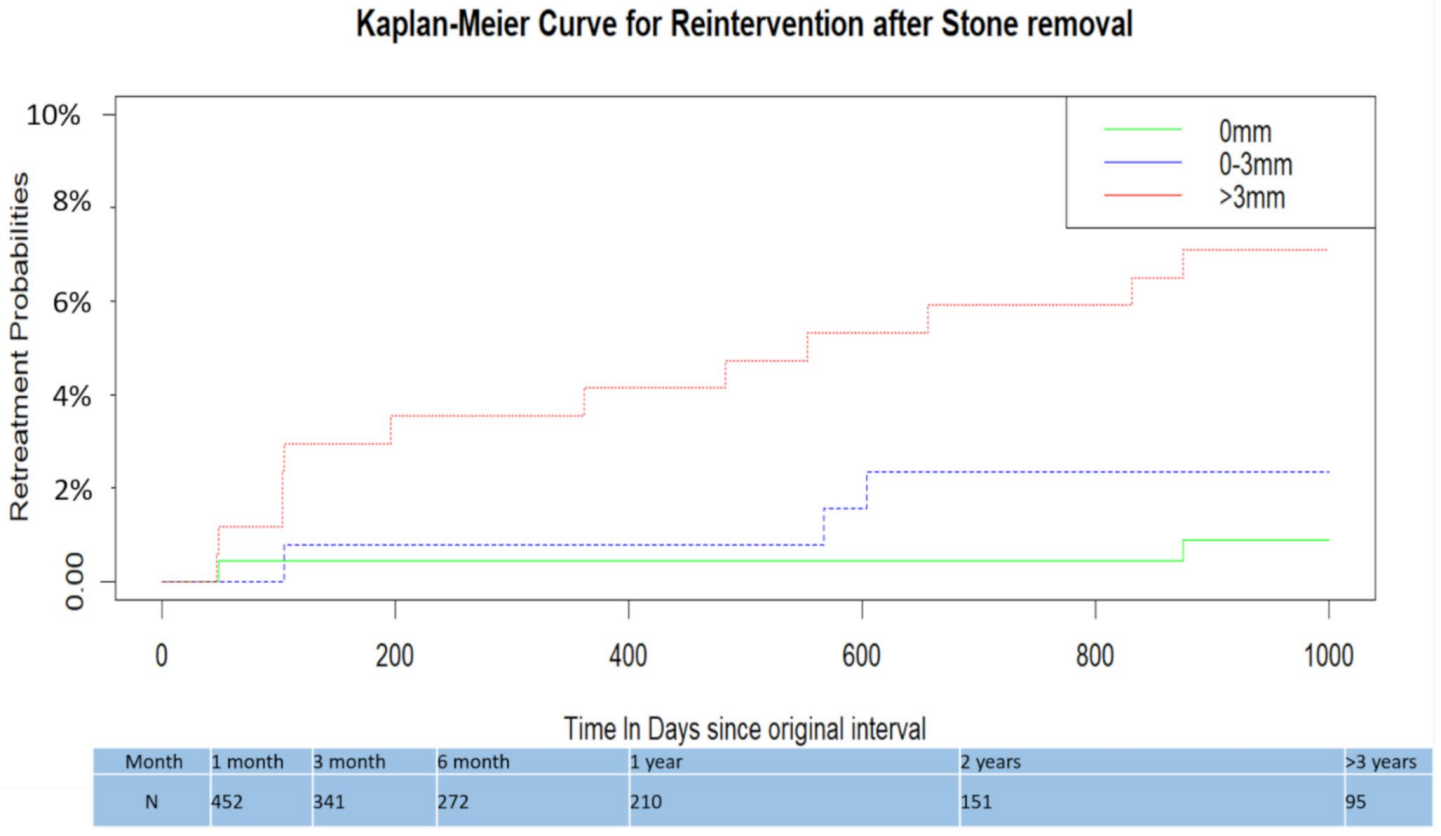
Kaplan-Meier Curve showing time to retreatment based on size of residual stone fragments as seen on post-ureteroscopy NCCT scan (N = number of patients available for postoperative follow-up time in months)

At a median follow-up of 26 months (IQR: 12.6–36 months; range: 1–70 months), the RTR demonstrated an association with RF size. Patients without identifiable RF had an RTR of 0.009%. However, it increased significantly to 2.3% in patients with RF *≤* 3 mm and further to 7.02% in those with RF > 3 mm (*p* < 0.001) (Fig. 3). Logistic regression analysis revealed that the IRR for retreatment in patients with RF > 3 mm compared to RF < 3 mm is approximately 7.14 (95% CI: 1.96–24.39). Moreover, several factors were identified as predictors of retreatment during follow-up. Preoperative stenting (*p* = 0.045) and larger preoperative stone size (*p* = 0.015) were significantly associated with an increased risk of retreatment (Fig. 3). Conversely, factors associated with a reduced risk of reintervention included achieving SFs (defined as RF < 3 mm) after surgery (*p* = 0.017) and higher BMI (*p* = 0.028) (Fig. 3). The Kaplan-Meier curve illustrated a proportional increase in retreatment rates with increasing size of RF sizes (Fig. 4). Results of the literature review of all studies evaluating SFR after ureteroscopy with CT scan are detailed in Table 2.

**Table 2:** Literature review of studies defining SFR after ureteroscopy based on postoperative NCCT

Author, year, Country, Study type	Sample size (Patient / RU)	Preoperative stone characteristics			SFR definition	Procedure-to-CT Interval*	Overall SFR	Significant Predictor of SFR
		Size of largest stone (mm)^1^	Stone/RU^1^	Location				
Pearle et al., 2005 USA, RCT [11]	32 patients	35.9 ± 18.4 mm^2^ (surface area)	NA	Lower pole	Complete absence	3 months	50% (0 mm) 72% (< 4 mm)	NA
Portis et al., 2006 USA, Prospective study [12]	58 patients	9.4 ± 3.4	NA	Ureter- 23 pt Kidney- 35 pt	0 mm (5 mm interval slice)	1 month	54% (0 mm) 83.9% (2 mm) 94.6% (4 mm)	SRF- 52.%- LC stone 5 6.3% non-LC renal stone 52.2% for ureter stone SFR not affected by location or multiplicity.
Macejko et al., 2009, USA Retrospective study [13]	92 patients, 113 renal units	8 ± 5	1.87	Kidney − 51% Ureter − 34% Both − 15%	Complete absence	Mean: 3 months Range: 1 day − 16.9	62.8% (*≤* 2 mm) 84.1% (< 4 mm) 50.4% (0 mm)	SFR- Kidney- 34.8% Ureter- 80% Both 29% Ureteric stones, single stones associated with higher SFR as compared with renal stone and multiple stones
Rippel et al., 2013 USA [14]	248 patients, 265 renal units.	7.6	1.4	Kidney – 30% Ureter – 50% Both – 20%	< 2 mm	Range: 1 to 3 months	62% (< 2 mm) 49% (< 1 mm)	SFR- Kidney- 48% Ureter- 80% Target stone 6–10 mm and > 10 mm were associated with lower SFR Ureteric stones associated with higher SFR (80% vs. 48%)
York et al., 2018 USA, Retrospective study [15]	214 patients, 288 renal units	6.2 (range 1–20).	6.4.	99% Renal Stones, 1% UPJ stones.	Complete absence*	Median: 1 (range of 0–3 months)	Low dose CT group: 76% Normal dose CT: 62%	NA
Danilovic et al., 2019 Brazil [16]	92 patients, 115 Renal units	7.92 ± 3.81. Stone size sum average: 14.92 ± 7.26	NA	100% Ureteric stones.	Complete absence	3 months	74.8% (0 mm) 83.5% (< 2 mm)	NA
Danilovic et al., 2019 Brazil [17]	94 patients; 1/4th of standard fluoroscopy dose group	10.6 ± 3.7	NA	100% Ureteric stones.	Complete absence	3 months	93.6%	NA
	Standard fluoroscopy dose group	10.2 ± 3.6					91.5%	
Johnson et al., 2020 USA Prospective study [18]	168 patients, 210 renal units.	16 ± 13 mm	Median: 2 (IQR 1–4)	NA	Not reported (NR)	6–8 weeks post procedure.	55%	Greater number of CaOx-Dihydrate stone composition predict lower SFR on multivariate A mean of 44 ± 37 ureteroscope passes
Yazici et al., 2024 Turkey, Prospective study [19]	178 patients	1212 ± 326 mm3 (volume) 17.58 ± 9.92	NA	100% kidney		4 weeks	< 4 mm 79.2% < 2 mm- 64% 0 mm- 56.7%	Ultrasound has high specificity but low sensitivity for SFR evaluation. Adding KUB does not improve diagnostic evaluation

(RCT: Randomized Clinical Trial; RU: Renal unit; ^**1**^Mean; *Patients with residual stones which corelated with intraoperative mapping area of pitting, plugging and tissue calcification were considered stone free; #- abstract)

## Discussion

Using postoperative NCCT, we observed that the definition of “stone-free” significantly impacts SFR determination after ureteroscopy. As the RF cut-off size increased, SFR improved, consistent with prior studies [11–14, 16, 19]. Our study’s true SFR was 42.8%, much lower than the up-to-90% rates cited in guidelines relying on less sensitive imaging like ultrasound (US) or x-ray. The sensitivity of the US to detect renal stones varies widely, ranging from 45 to 54% [20, 21]. In comparison, SFRs determined by CT, US, and x-ray KUB were 56.7%, 76.4%, and 89.8%, respectively [19]. Imaging timing also plays a significant role. While most authors performed CT within 1–3 months post-ureteroscopy (Table 2), our NCCT was conducted at 3–4 weeks. Earlier imaging may detect transient fragments, reducing observed SFR. EAU guidelines recommend imaging at 4 weeks to allow for spontaneous fragment passage [7].

Pearle et al. first reported the concept of true SFR using postoperative NCCT at 3 months after ureteroscopy in 2005 when authors noted true SFR of 50% for lower pole renal calculi. This inferior SFR was initially attributed to stone location [11]. However, other studies similarly reported lower SFRs of 34.8–56.7% for renal calculi over the last two decades, despite technological advances [13, 19, 22]. Our findings align with prior CT-based studies. It is possible that RF observed in our series may include renal calcifications or Randall’s plaques, which NCCT cannot distinguish from true stones. If ureteroscopic mapping of nephrocalcinosis was performed and these parenchymal calcifications were excluded from our calculation, the SFR would likely be higher. York et al. employed this strategy and noted that 73% of their renal units were SF on NCCT [15]. Unfortunately, the number of patients with renal calculi on postoperative CT scans confirmed to be nephrocalcinosis (and not RF) during ureteroscopy was not specified in their study.

In our study, decreased SFR was linked to positive preoperative urine culture, prior nephrolithiasis treatment, larger stone size, and mid-pole stone location. Our baseline stone size was higher than most studies (Table 2). Patients with prior surgical treatments, particularly PCNL, had higher RF risks, likely due to altered renal anatomy or stone location [23]. Preoperative stents and lower/mid-pole stones also contributed to lower SFR. Lower pole stones are challenging to remove, though we attempted to relocate them before laser lithotripsy as it improves outcomes compared to in-situ treatments [24]. This might be a possible reason why mid-pole stone location and not lower pole location was noted to be statistically significant factor for lower SFR on multivariate analysis. Single stones in the ureter were associated with higher SFR than multiple stones in our study.

Johnson et al. reported increased SFR on postoperative NCCT with fewer stones, smaller aggregate size, and middle pole location. In comparison, Rippel et al. found persistent RF on postoperative NCCT risks linked to larger stones, kidney/ureter location, multiple calculi, and longer operative time [11, 14]. Our findings align with these studies, except operative time did not significantly affect SFR in our cohort. Pre-stenting was surprisingly associated with decreased SFR, potentially due to its use in larger, more complex stones or cases of urosepsis. Positive urine culture was also associated with lower SFR, likely influencing intraoperative decisions to reduce sepsis risk. While SFR for ureteral stones was higher than renal calculi, the difference was not statistically significant [12–14].

We noted that reintervention rates after URS were 7% at a mean follow-up of 26 months (IQR: 12.6–36 months) for patients with RFs > 3 mm, compared to almost none for those with no RF on postoperative NCCT. Larger RF size is a well-established risk factor for reintervention, resulting from symptomatic passage or growth of fragments [3, 13]. Previous stent placement was also associated with higher reintervention rates in our cohort, likely due to the link with decreased SFR. Our rate is notably lower than the 19% retreatment rate within three months, as reported in a 2014 insurance database of over 25,000 patients [25]. Similarly, a multicenter study found a retreatment rate of 29% at 16.76 months for patients with RF present on imaging within 12 months [2]. A systematic review of 2,096 patients noted higher reintervention rates for RF > 4 mm compared to < 4 mm (22% vs. 47% at 50 months) [26]. Only two ureteroscopy studies used NCCT to define RF after ureteroscopy and reported RTR; Rebuck et al. reported a 19.6% reintervention rate at 1.6 years, while Ozgor et al. found 25% of patients with < 5 mm RF required reintervention at 30.5 months [26, 27]. Our lower rates might be attributable to planned two-stage ureteroscopy for patients with large stone burdens (> 2 cm) and the exclusion of chronic nephrocalcinosis cases. Notably, post-op NCCT is known to identify significantly more RF ≥ 4 mm (35.7% vs. 13.9%), potentially inflating retreatment rates for these patients [28]. European Association of Urology Guideline Panel on Urolithiasis recommends at least one CT scan to assess RF burden and plan reinterventions accordingly, with treatment advised for RF > 4 mm due to high retreatment risks [29]. Hence, we selected definition of residual fragments < 3 mm for our analysis.

The inherent radiation exposure related to post-ureteroscopy NCCT needs to be discussed. To limit the radiation exposure, we employed low-dose NCCT scans after ureteroscopy at our institution. The approximate radiation exposure at our institution during NCCT is 1.5-2mSv. A systematic review showed that both low-dose have diagnostic accuracy of 95% compared to standard-dose NCCT scans despite a significant reduction in radiation dosage [28]. Most newer studies report an average radiation dose from 1 to 1.5 millisieverts for low-dose NCCT scans, which is marginally more than the average radiation dose from an x-ray KUB (0.5–1.1 mSv) [30].

Our study carries limitations inherent to the retrospective design and the risk of not capturing all stone-related outcomes during the follow-up period. Not all patients were stented after ureteroscopy and duration of post-operative stenting varied from 5 days to 1 month. This might influence SFR. Despite this limitation, to our knowledge, our study is the largest single-institution study to date on evaluating factors responsible for CT-guided SFR after ureteroscopy in the real world and on the influence of CT-detected RFs on reintervention rates.

## Conclusions

The true SFR based on postoperative NCCT scan was 42.8%. On multivariate analysis, positive preoperative urine culture, a prior history of urolithiasis treatment, increasing stone size, and mid-pole stone location negatively influenced SFR. The size of RFs strongly correlated with the RTR, which was 7% at a mean follow-up of 26 months for patients with stones > 3 mm and almost none for patients with no RF on postoperative CT. Hence, all attempts should ideally be made to render the patient completely stone-free after ureteroscopy.

## Data Availability

The datasets generated and/or analyzed during the current study are available from the corresponding author upon reasonable request.
